# Identification of four key biomarkers and small molecule drugs in nasopharyngeal carcinoma by weighted gene co-expression network analysis

**DOI:** 10.1080/21655979.2021.1949844

**Published:** 2021-07-14

**Authors:** Xi Pan, Jian-Hao Liu

**Affiliations:** a Department of Oncology, Xiangya Third Hospital, Central South University, Changsha, China; b School of Pharmaceutical Sciences of Central South University, Changsha, 410078, China

**Keywords:** Nasopharyngeal carcinoma, hub genes, WGCNA, signature, small-molecule drugs

## Abstract

Nasopharyngeal carcinoma (NPC) is a heterogeneous carcinoma whose underlying molecular mechanisms involved in tumor initiation, progression, and migration are largely unclear. The aim of the present study was to identify key biomarkers and small-molecule drugs for screening, diagnosing, and treating NPC via gene expression profile analysis. Raw microarray data was used to identify 430 differentially expressed genes (DEGs) in the Gene Expression Omnibus (GEO) database. The key modules associated with histological grade and tumor stage were identified using weighted gene co-expression network analysis. qRT-PCR was used to verify the differential expression of hub genes. Gene ontology (GO), Kyoto Encyclopedia of Genes and Genomes (KEGG), and the connectivity map database were used to identify potential mechanisms and screen small-molecule drugs targeting hub genes. Functional enrichment analysis showed that genes in the green module were enriched in the regulation of cell cycle, p53 signaling pathway, and cell part morphogenesis. Four DEG-related hub genes (*CRIP1, KITLG, MARK1*, and *PGAP1*) in the green module, which were considered potential diagnostic biomarkers, were taken as the final hub genes. The expression levels of these four hub genes were verified via qRT-PCR, and the results were consistent with findings from the GEO analysis. Screening was also conducted to identify small-molecule drugs with potential therapeutic effects against NPC. In conclusion, four potential prognostic biomarkers and several candidate small-molecule drugs, which may provide new insights for NPC therapy, were identified.

## Graphical Abstract

**Figure:**
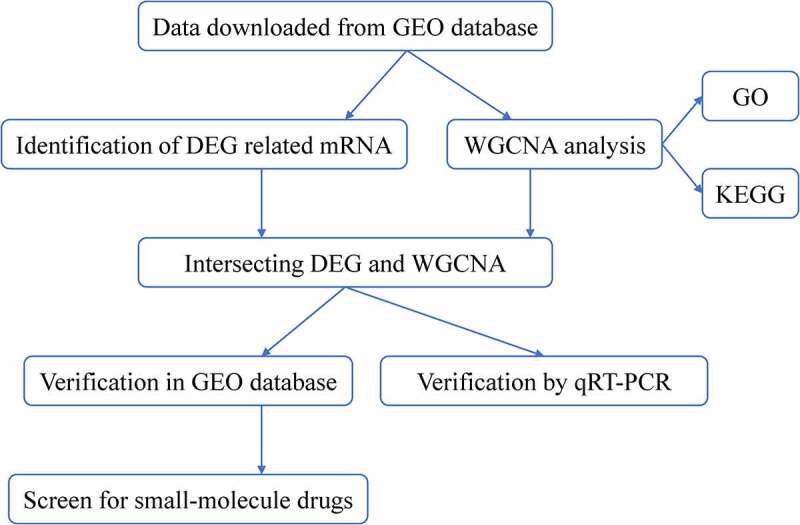

## Introduction

Nasopharyngeal carcinoma (NPC) is a malignant carcinoma arising from the nasopharyngeal epithelial lining of the nasopharynx. It has a unique geographical distribution and racial prevalence. Based on GLOBOCAN cancer estimates and the International Agency for Research on Cancer, there were approximately 129,079 newly diagnosed NPC cases and 72,987 deaths in 2018, accounting for approximately 0.7% of cancer cases and deaths in that year [1,2]. The geographical distribution of global NPC incidence is extremely unbalanced; more than 80% of newly diagnosed cases are in Asia, while fewer than 0.2% of the cases occur in Oceania, resulting in an age-standardized rate of 10.0 per 100,000 persons in Southeast Asia and 0.5 per 100,000 persons in Central America [1,2]. Despite substantial improvements in NPC treatment options over the past decade, including chemotherapy, immunotherapy, and radiotherapy, the prognosis for patients with NPC remains low; approximately 20% of cases recur and the 5-year survival rate is only 50%–70% [3]. Thus, novel biomarkers and therapeutic strategies are urgently required for effective diagnosis and improved treatment for NPC patients. The remarkable geographical distribution and racial prevalence of NPC incidence has spurred studies on its risk factors, and it is thought that several etiological factors, including genetic and ethnic factors, Epstein-Barr virus (EBV) infection, and environmental factors (e.g. cigarette smoking and exposure to dust and formaldehyde) contribute to the development and progression of NPC [4–8]. EBV infection is perhaps the most extensively studied etiological factor in NPC pathogenesis. The study involves in situ hybridization of EBV-encoded RNA, which is found in all tumor cells but not in the normal nasopharyngeal epithelial lining of the nasopharynx, suggesting that EBV activation may play a vital role in the pathogenesis of NPC [9–11]. There is growing evidence that EBV is associated with multiple human cancers, including NPC [7–10]. Apart from EBV infection, genetic susceptibility is perhaps the most common cause of NPC. Studies have identified genetic susceptibility genes involved in DNA repair, and metabolic and immune response pathways are associated with the development of NPC [12–14]. Recent linkage and association studies have described a potential association between genetic susceptibility genes and the development of NPC, including *MST1R, PTPRG, HLA*, and *MMP2* [12–17]. The rapid developments in high-throughput whole-genome sequencing and bioinformatic analyses have played a significant role in the screening for candidate biomarkers of several diseases, including NPC.

In this study, we sought to obtain relevant data from the Gene Expression Omnibus (GEO) database and undertook a weighted gene co-expression network analysis (WGCNA) to identify key modules. We then explored the underlying mechanisms of hub module genes using gene ontology (GO) and Kyoto Encyclopedia of Genes and Genomes (KEGG). Based on the connectivity map database, four potential small-molecule drugs targeting the hub genes were identified. Two NPC cohorts from the GEO database and experimental data from our study were used to verify the expression levels of these four hub genes. The aim of our study was to identify key biomarkers and validate the expression levels of four hub genes, and to further screen for small-molecule drugs of these hub genes.

## Materials and methods

### Microarray datasets

Five mRNA microarray datasets (GSE12452, GSE53819, GSE13597, GSE40290, and GSE64634) (Table 1) were downloaded from the GEO database (https://www.ncbi.nlm. nih.gov/geo/), a public functional genomics data repository that includes array- and sequence-based gene profile data [18].

**Table 1.** Primers used for the qRT-PCR

MARK1	F: 5‘- GAGCGGGACACGGAAAATCAT −3’
	R: 5‘- TGCTACTCGACTTGGTAGGCT −3’
PGAP1	F: 5‘- CTTCGGCTTCGAGGAGAATAAG −3’
	R: 5‘- GGGATAGCGTTTTGCCAGTTT −3’
KITLG	F: 5‘- AATCCTCTCGTCAAAACTGAAGG −3’
	R: 5‘- CCATCTCGCTTATCCAACAATGA −3’
CRIP1	F: 5‘- TAATGACGGCCCGGTCTTTTA −3’
	R: 5‘- TGCAGCGTGCTGGGTTTAAT −3’
GAPDH	F: 5‘- TGTGGGCATCAATGGATTTGG −3’
	R: 5‘- ACACCATGTATTCCGGGTCAAT −3’

### Data processing and identification of differentially expressed genes (DEGs)

The robust multiarray averaging (RMA) method was used for data pre-processing, including background adjustment, quantile normalization, and log2 transformation of expression matrices. The corresponding platforms were used to annotate each probe based on the Entrez ID. For genes with several probes, the medians of all probes were selected. The ‘limma’ package in the R statistical software (version 3.6) was used to identify differentially expressed genes associated with NPC, with |logFC| >1 and *P-*value < 0.05 set as the screening thresholds.

### Construction of weighted gene co-expression networks

The median absolute deviation of each gene expression value was calculated and genes with median absolute deviation in the top 25% were chosen for WGCNA. The ‘WGCNA’ package in R was used to construct weighted gene co-expression networks [19]. First, the goodSamplesGenes function was used to detect good samples and good genes. Second, the pickSoftThreshold function was used to select an appropriate soft-thresholding power. Then, the co-expression similarity was raised to achieve a scale-free topology based on the appropriate soft-threshold power. Finally, cox-expression gene modules were generated using the cutreeDynamic function. Additionally, we calculated gene significance (GS), module eigengene (ME), and module membership (MM) to detect the association between clinical parameters of either the genes or modules; *P* < 0.05 was considered statistically significant.

### Functional enrichment analysis of hub module genes

To better understand the potential molecular mechanisms by which hub module genes impact correlative clinical parameters, we uploaded all hub module genes to the Metascape online tool (https://metascape.org/gp/index.html#/main/step1) [20] where Gene ontology (GO) and Kyoto Encyclopedia of Genes and Genomes (KEGG) pathway enrichment analyses were performed. *P* < 0.01 was considered statistically significant.

### Selection and validation of hub genes

Genes with the highest intra-modular connectivity were selected as candidate hub genes. Genes with biological significance usually have higher absolute GS values. In this study, candidate hub genes were screened based on the criteria: absolute GS value > 0.20 and MM > 0.80. The final hub genes were identified from the intersection of NPC-related DEGs with candidate hub genes. Expression levels of hub genes in patients with NPC were assessed using box plots. The diagnostic values of hub genes were evaluated using the area under the receiver operating characteristic (ROC) curve. Differential expression and diagnostic values of hub genes were validated in NPC patients using a separate external dataset (GSE53819). Three microarray datasets (GSE13597, GSE40290, and GSE64634) were then combined to further verify the expression levels of hub genes.

### Identification of candidate small-molecule drugs

The connectivity map (CMAP) is an online database (https://portals.broadinstitute.org/cmap) with more than 7000 gene expression profiles of 1309 compounds that is used to predict small-molecule drugs for specific diseases [21]. To identify small-molecule target drugs for treating NPC, the hub genes were uploaded to the CMAP database’s ‘query’ page. Correlation between the small-molecule drugs and hub genes was evaluated using enrichment scores ranging from −1 to 1. The chemical and molecular structures of the small-molecule drugs were downloaded from the PubChem Compound database (https://pubchem.ncbi.nlm.nih.gov/).

### Tissue collection

NPC specimens and adjacent nasopharyngeal epithelial tissues were collected from 20 patients, immediately transferred into liquid nitrogen and then preserved at −80°C. This study was approved by the Ethics Committee of the Xiangya Third Hospital, Central South University and was conducted in accordance with the Declaration of Helsinki.

### Cell culture

The human immortalized nasopharyngeal cell line, NP69, was purchased from the American Tissue Culture Collection (ATCC; Manassas, VA, USA) and human nasopharyngeal carcinoma cell lines (CNE1, HNE3, 5–8 F, and HK-1) were purchased from the cell bank of the Chinese Academy of Sciences in Shanghai (Shanghai, China). The NP69 cell line was cultured in keratinocyte serum-free medium (KSFM; Gibco, Grand Island, NY, USA) containing 10%–15% fetal bovine serum (FBS; Gibco), 100 U/ml penicillin, and 100 μg/mL streptomycin and incubated in a saturated humidity incubator set at 37°C and 5% CO^2^. CNE1, HNE3, 5–8 F, and HK-1 cells were cultured in Dulbecco’s Modified Eagle’s Medium (DMEM; Gibco, Grand Island, NY, USA) containing 10%–15% FBS, 100 U/ml penicillin, and 100 μg/mL streptomycin and incubated in a saturated humidity incubator set at 37°C and 5% CO^2^.

### RNA extraction and quantitative reverse transcription PCR (qRT-PCR)

RNA was extracted from the cells using TRIzol reagent and reverse transcribed into cDNA using the following reaction mixture: 2 μL cDNA, 10 μL SYBR® Premix Ex Taq^TM^ 11 (Takara Bio, Tokyo, Japan), 0.4 μL forward and reverse primers, and 6.4 μL ddH2O. Each sample was run in triplicate and GAPDH was used as the internal control. The reaction conditions used were: pre-denaturation at 95°C for 10 min followed by 40 cycles of denaturation at 95°C for 15 s, annealing at 60°C for 30 s, and extension at 70°C for 30 s. The data was analyzed using the ΔΔCT method and the average of the three replicate experiments for each sample was taken. The primers used are shown in Table 2.

**Table 2.** Characteristics of datasets included in the integrated analysis

**Year**	**Source**	**First author**	**Country**	**Assay type**	**Platform**	**PMID**
2008	GSE12452	Ahlquist P	USA	Affymetrix Human Genome U133 Plus 2.0 Array	GPL570	22,880,099
2014	GSE53819	Qian CN	China	Agilent-014850 Whole Human Genome Microarray 4x44K	GPL6480	24,763,226
2009	GSE13597	Yap L	United Kingdom	Affymetrix Human Genome U133A Array	GPL96	19,142,888
2016	GSE40290	Huang ZX	China	Capitalbio 22 K Human oligo array version 1.0	GPL8380	NA
2017	GSE64634	Xiong W	China	Affymetrix Human Genome U133 Plus 2.0 Array	GPL570	26,246,469

### Statistical analysis

Two-tailed Student’s t-test was used to identify differentially expressed genes associated with NPC. Statistical analyses were conducted in R (version 4.0.0), MedCalc (version 19.1) or SPSS (version 24.0) software. Genes with |logFC| >1 and *P* < 0.05 were considered statistically significant DEGs.

## Results

This study used WGCNA to identify key gene modules associated with NPC histological grade and tumor stage. Genes with the highest intra-modular connectivity were selected as candidates. These hub genes were identified from the intersection of NPC-related DEGs with candidate hub genes. GO and KEGG analyses were performed to explore the mechanisms underlying the hub module genes. The connectivity map database was used to screen for potential small-molecule drug targets of the hub genes. Finally, two NPC cohorts from the GEO database were used to verify the expression levels of the four hub genes.

### Identification of DEGs

**Figure 1.**
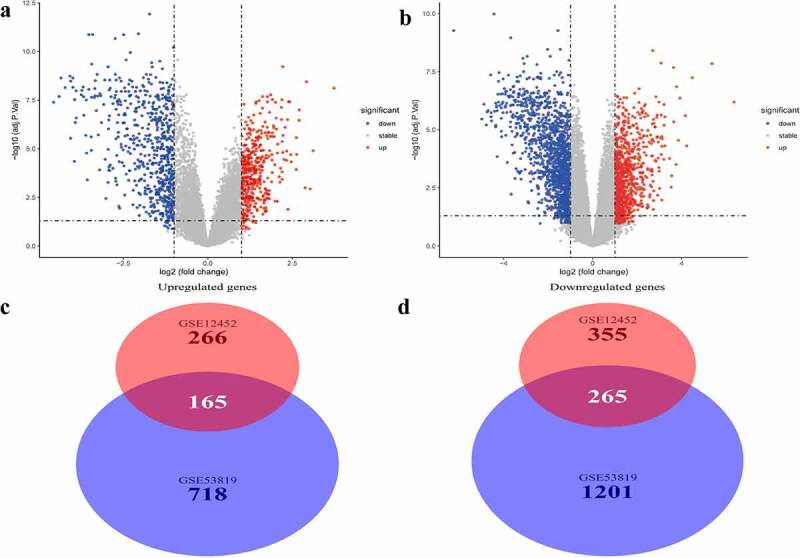
Differentially expressed genes and common differentially expressed genes in two datasets. (a and b) The volcano plots visualize the differentially expressed genes in GSE12452 and GSE53819. (c and d) Common differentially expressed genes in two datasets

Two microarray datasets, GSE12452 and GSE53819, from the GEO database, were used to identify DEGs between normal samples and NPC samples. DEGs were identified based on the thresholds |logFC| >1 and *P* < 0.05. Volcano plots of DEGs in the two separate datasets are shown in Figure 1a **and** Figure 1b. A total of 430 common genes were identified at the intersection of DEGs in the two datasets for further WGCNA. Of the 430 genes, 165 were significantly upregulated while 265 were significantly downregulated in NPC patients (Figure 1c **and** Figure 1d).

### Construction of weighted gene co-expression network and identification of hub modules

**Figure 2.**
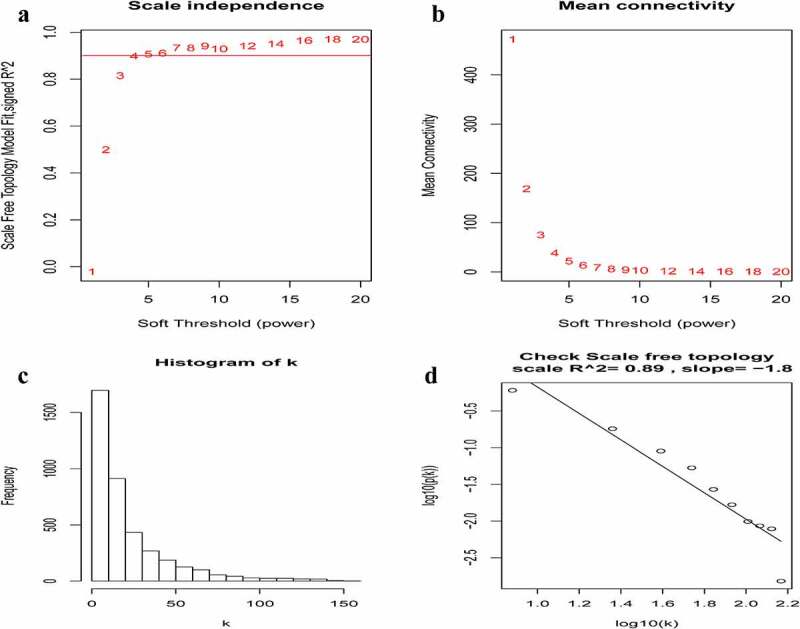
Determination of soft-thresholding power in the weighted gene co-expression network analysis (WGCNA). (a) Analysis of the scale-free fit index for various soft-thresholding powers (β). The red line indicates where the correlation coefficient is 0.9, and the corresponding soft-thresholding power is 5. (b) Analysis of the mean connectivity for various soft-thresholding powers. The red line indicates where the correlation coefficient is 0.9, and the corresponding soft-thresholding power is 5. (c) Histogram of connectivity distribution when β = 5. (d) Checking the scale-free topology when β = 5

**Figure 3.**
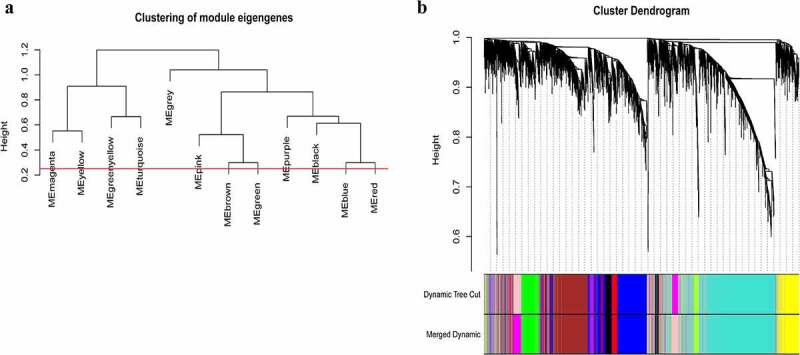
Construction of WGCNA modules. (a) The cluster dendrogram of module eigengenes. (b) The cluster dendrogram of the genes with median absolute deviation in the top 25% in the GSE12452. Each branch in the figure represents one gene, and every color below represents one co-expression module

**Figure 4.**
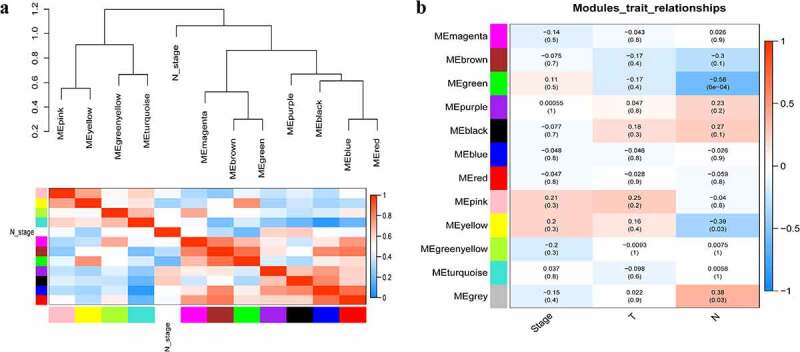
Relationship between modules and clinical traits. (a) Module eigengene dendrogram and eigengene network heatmap summarize the modules yielded in the clustering analysis. (b) Heatmap of the module-trait relationships. The green module was significantly associated with N stage

A total of 3949 genes with median absolute deviations in the top 25% in 31 NPC samples and 10 normal samples in GSE12452 dataset were selected. The samples were clustered to remove outlier samples. WGCNA was then used to identify genes that were highly co-expressed across the groups. To ensure a scale-free network, the power of β = 5 (scale-free R^2^ = 0.89, slope = −1.80) was selected as the soft-thresholding, as shown in Figure 2. Then, similar modules were merged based on the height of ME in the clustering equal to 0.25 (Figure 3a). 12 co-expressed gene modules were identified. The ‘grey’ module, which contained 583 genes not attributed to any modules (Figure 3b), was removed in the subsequent analysis. The relationships among the 11 modules were analyzed using clinical parameters (including histological grade and tumor stage). The green module was significantly associated with NPC tumor stage (N stage) and was chosen as the clinically significant module for subsequent analyses (Figure 4).

### Functional enrichment analysis of hub module genes

**Figure 5.**
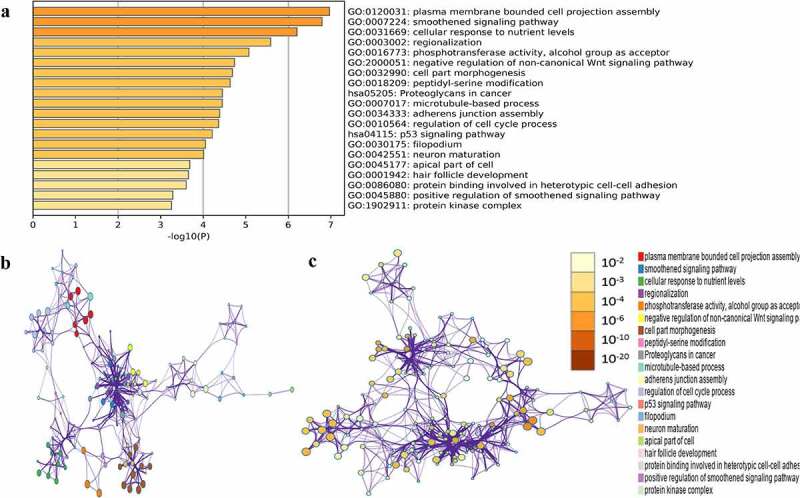
Functional enrichment analysis of hub module genes. (a) GO terms and KEGG pathway were presented, and each band represents one enriched term or pathway colored according to the -log10(p). (b) Network of the enriched terms and pathways. Nodes represent enriched terms or pathway with node size indicating the number of hub module genes involved in. Nodes sharing the same cluster are typically close to each other, and the thicker the edge displayed

GO and KEGG pathway enrichment analysis of the above green module was carried out to mine potential biological functions associated with NPC using the Metascape online tool. *P* < 0.01, minimum overlap genes = 3, and an enrichment factor > 1.50 were set as the cutoff criteria. GO analysis showed that genes in the green module were mainly enriched in plasma membrane-bound cell projection assembly, smoothened signaling pathway, cellular response to nutrient levels, regulation of cell cycle process, neuro maturation, and protein kinase complex (Figure 5). Two significantly enriched pathways, the p53 signaling pathway and proteoglycans in cancer, were associated with genes in the green module.

### Identification and validation of hub genes

**Figure 6.**
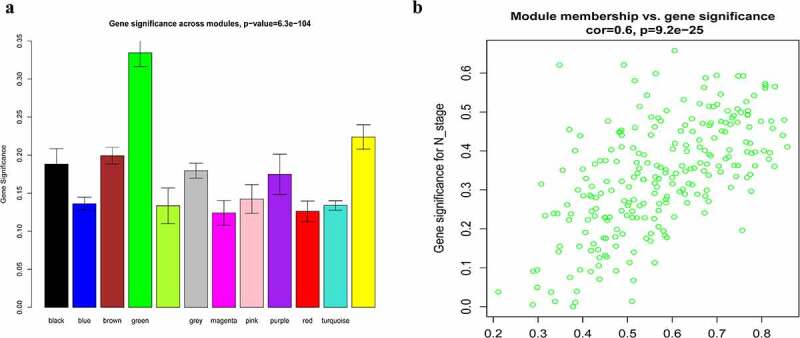
Identification of hub module and candidate hub gene. (a) Distribution of average gene significance and errors in the modules associated with the NPC. (b) Scatter plot for correlation between gene module membership in the green module and gene significance

**Figure 7.**
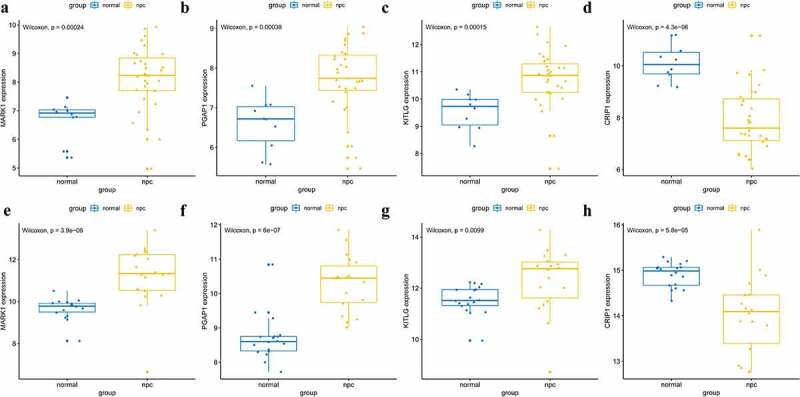
Validation of hub genes in the gene expression level. (a-d) Validation of hub genes in the GSE12452. PGAP1, MARK1, and KITLG were significantly higher expression in NPC compared with normal tissues, while CRIP1 was significantly lower expression in NPC compared with normal tissues. (e-h) Validation of hub genes in the GSE53819 and the results were the same as earlier

**Figure 8.**
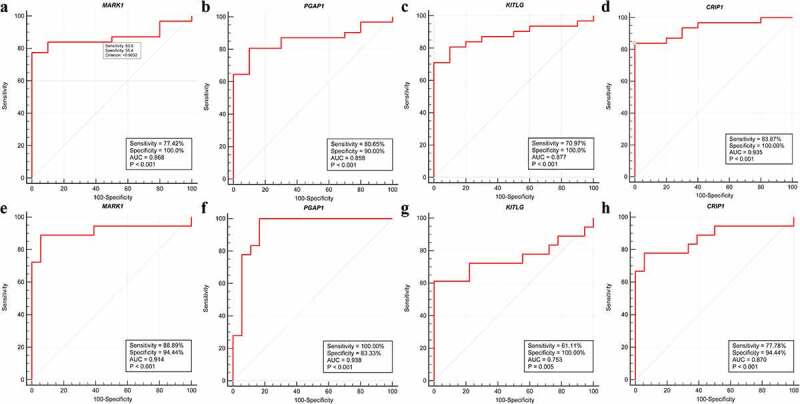
Validation of hub genes in the diagnostic value. (a-d) Validation of hub genes in the GSE12452. Receiver operating characteristic (ROC) curves and area under the curve (AUC) statistics are used to evaluate the capacity to discriminate NPC from normal controls with excellent sensitivity and specificity. (e-h) Validation of hub genes in the GSE53819 and the results were the similar as earlier. These findings indicated these four hub genes have excellent diagnostic efficiency in NPC

**Figure 9.**
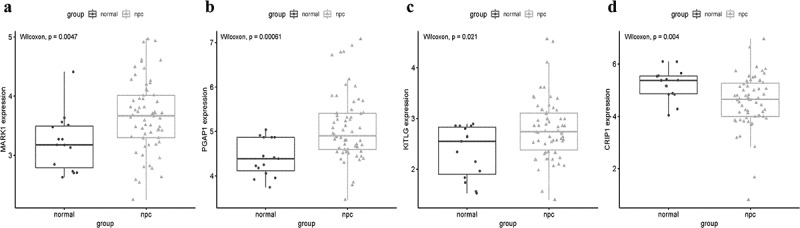
Validation the expression levels of four hub genes in the combined microarray datasets. (a-d) PGAP1, MARK1, and KITLG were significantly higher expression in NPC compared with normal tissues, while CRIP1 was significantly lower expression in NPC compared with normal tissues

A total of 14 genes with the greatest connectivity in the green module were identified as candidate hub genes based on GS > 0.2 and MM > 0.8 (Figure 6). The final four hub genes: cysteine-rich intestinal protein 1 (*CRIP1*), KIT ligand (*KITLG*), microtubule affinity regulating kinase 1 (*MARK1*), and post-GPI attachment to protein 1 (*PGAP1*), were identified from the intersection of NPC-related DEGs with candidate hub genes. Data in the GSE12452 and GSE53819 datasets were compared with that of normal tissues, and three of the final four hub genes: *PGAP1, MARK1*, and *KITLG* showed significantly higher expression in NPC tissues, while the expression of *CRIP1* was significantly downregulated (Figure 7). To verify the diagnostic potential of these four hub genes, ROC analysis was performed on the GSE12452 and GSE53819 datasets. The results of the ROC analysis demonstrated that these four genes had excellent diagnostic efficiencies in NPC patients (Figure 8). The expression levels of the four hub genes were further validated in the combined microarray datasets (Figure 9).

### Identification of potential small-molecule drugs for NPC treatment

**Figure 10.**
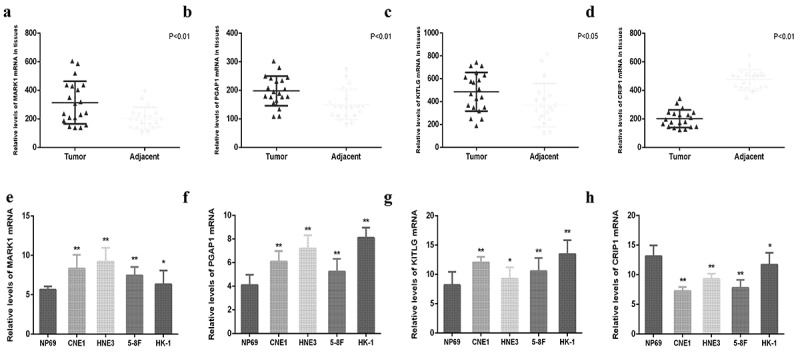
Relative expression of MARK1, PGAP1, KITLG and CRIP1 mRNA in clinical nasopharyngeal carcinoma tissues and cell lines. (a-d) The relative expression of MARK1, PGAP1, KITLG and CRIP1 mRNA in 20 paired nasopharyngeal carcinoma tissues and adjacent tissues were detected by qRT-PCR. (e-h) The relative expression of MARK1, PGAP1, KITLG and CRIP1 mRNA in 5 cell lines (NP69, CNE1, HNE3, 5–8 F and HK-1) were detected by q-RT-PCR. *P < 0.05, **P < 0.01, ***P < 0.001

The hub genes were uploaded to the CMAP database to identify potential small-molecule drugs for NPC treatment. Based on the criteria: number of instances n > 5 and *P* < 0.05, the top ten most significant small-molecule drugs, which might be potential therapeutic targets for NPC treatment, were identified to be vorinostat, pioglitazone, fludrocortisone, thalidomide, bendroflumethiazide, chlorcyclizine, etiocholanolone, hyoscyamine, sulfadiazine, and biperiden (Table 3 and Figure 10).

**Table 3.** Top 10 most significant small molecule drugs based on CMAP

**CMAP name**	**Mean**	**Number**	**Enrichment**	***P*-value**	**Specificity**	**Percent non-null**
vorinostat	0.369	12	0.423	0.0171	0.6181	58
pioglitazone	0.234	11	0.412	0.03326	0.2313	54
fludrocortisone	−0.265	8	−0.469	0.03975	0.3732	50
thalidomide	0.497	7	0.607	0.00501	0	71
bendroflumethiazide	0.33	6	0.598	0.01363	0.0145	50
chlorcyclizine	−0.486	6	−0.592	0.01619	0.0089	66
etiocholanolone	−0.481	6	−0.581	0.01903	0.2208	66
hyoscyamine	0.62	5	0.717	0.00411	0	100
sulfadiazine	0.667	5	0.711	0.00473	0	100
biperiden	−0.354	5	−0.674	0.00863	0.1905	60

**Abbreviations**: CMAP, Connectivity map.

### Validation of the expression levels of four hub genes in NPC tissues and cell lines

**Figure 11.**
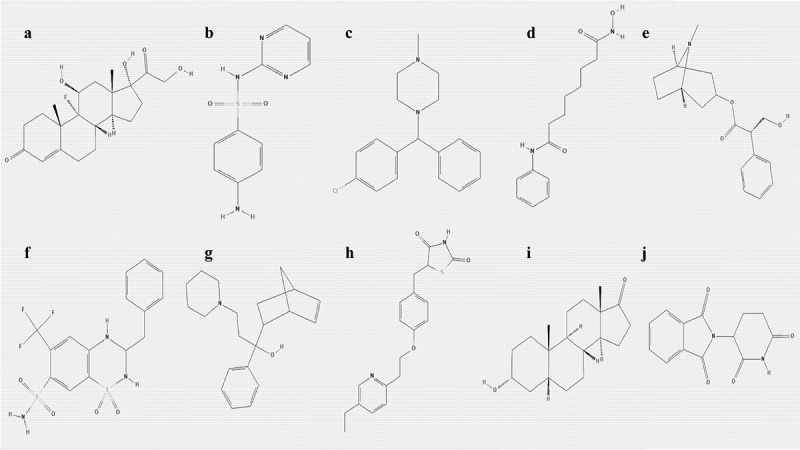
The 2D molecular structures of the top ten most significant small-molecule drugs. (a) Fludrocortisone. (b) Sulfadiazine. (c) Chlorcyclizine. (d) Vorinostat. (e) Hyoscyamine. (f) Bendroflumethiazide. (g) Biperiden. (h) Pioglitazone. (i) Etiocholanolone. (j) Thalidomide

The mRNA expression levels of *MARK1, PGAP1, KITLG*, and *CRIP1* genes in 20 pairs of NPC and adjacent tissues were quantified via qRT-PCR. The results showed that the expression of *MARK1, PGAP1*, and *KITLG* was upregulated in NPC samples relative to adjacent tissues (Figure 11(a-c)), while the expression of *CRIP1* was downregulated in NPC samples relative to adjacent tissues (Figure 11d). The expression of *MARK1, PGAP1, KITLG*, and *CRIP1* genes in four NPC cell lines (CNE1, HNE3, 5–8 F, and HK-1) and normal human immortalized nasopharynx cell line (NP69) were measured via qRT-PCR. The results showed that *MARK1, PGAP1*, and *KITLG* expression was upregulated in NPC cell lines relative to the normal human nasopharynx cell line (Figure 11(e-g)), while *CRIP1* expression was downregulated in NPC cell lines relative to normal human nasopharynx cell lines (Figure 11h).

## Discussion

Based on estimates from GLOBOCAN, there were 129,079 newly diagnosed NPC cases and 72,987 deaths reported globally in 2018 [1]. Etiologic factors associated with NPC include genetic, ethnic, EBV infection, and environmental factors [4–8]. Despite substantial improvements in the treatment of NPC over the past decade, the prognosis for patients with NPC diagnosis remain largely unsatisfactory due to a lack of obvious clinical signs in its early stages, with most initial diagnoses being made when the disease is at an advanced stage [3]. Therefore, elucidating the molecular mechanisms underlying NPC is of great significance.

WGCNA was used to identify potential molecular biomarkers and small-molecule drugs associated with NPC progression. We identified 430 DEGs and 11 co-expressed gene modules. The green module had the highest correlation with NPC tumor stage. Four genes, which had high functional significance in the clinically significant module, were identified as the final hub genes. Compared with normal tissues, *PGAP1, MARK1*, and *KITLG* showed significantly higher expression in patients with NPC, while *CRIP1* expression was significantly lower in patients with NPC in both the test and validation datasets. These results were confirmed via qRT-PCR. The results of the ROC analysis showed that these four hub genes had excellent diagnostic efficiency in tumor and normal tissues. Furthermore, functional enrichment analysis showed that green module genes were mainly enriched in cellular response to nutrient levels, regulation of cell cycle process, and p53 signaling pathway.

*PGAP1*, the gene encoding the post-GPI attachment to protein 1, is closely associated with the cell cycle process, cell proliferation, cell differentiation, and multiple signaling transduction pathways. *PGAP1* can directly and/or indirectly induce carcinogenesis through tumor cell proliferation, migration, and resistance to multiple drugs [22–25]. *MARK1*, also known as partitioning defective gene 1 (*Par-1*), is a member of the serine-threonine kinase family, which plays a vital role in the regulation of cell migration, cell proliferation, and establishment and maintenance of cell polarity and microtubules [26–28]. Several studies have revealed that *MARK1* plays an important role in tumorigenesis, and Natalia *et al* [29] found that *MARK1* is a novel functional target for miR-125a-5p, with implications in the regulation of stimulated cell migration of cervical tumor cell lines. Tang et al [30] showed that *MARK1* overexpression was correlated with cell migration and proliferation in colorectal cancer through its regulation of miR-23a expression. *KITLG*, encoding the ligand for the tyrosine kinase receptor encoded by the KIT locus, plays an essential role in the regulation of cell survival and proliferation, stem cell maintenance, mast cell development, and migration. KITLG/SCF binding can activate several signal transduction pathways [31–33]. *KITLG* gene expression levels were evaluated in testicular germ cell cancer, breast cancer, and gastrointestinal stromal tumors [34–37]. Overexpression of *KITLG* was correlated with increased tumor cell migration and proliferation, suggesting a tumorigenic role for *KITLG* [34,36]. *KITLG* was expressed at significantly higher levels in normal mammary gland epithelial tissue than in breast tumor tissue, indicating that it might be involved in the homeostasis of normal mammary tissue and its disruption would confer breast carcinogenesis [38]. CRIP1 is a member of the LIM/double zinc finger protein family, which is highly expressed in the intestine and immune cells [39]. In gastric cancer, *CRIP1* was overexpressed in primary tumor tissues and confirmed to be an independent prognostic factor. Patients with gastric carcinoma overexpressing *CRIP1* had shorter overall survival [40]. Compared with adjacent normal tissues, *CRIP1* was significantly more highly expressed in cervical cancer and was associated with FIGO stage [41].

The connectivity map database was used to screen small-molecule drugs with promising therapeutic capabilities against NPC. To screen out potential small-molecule drugs for treating NPC, 66 candidate small-molecule drugs were obtained from the CMAP database prediction based on the final NPC hub genes. Among the top ten most significant potential small-molecule drugs, vorinostat and thalidomide are particularly interesting and have anti-tumor effects in NPC treatment. Vorinostat is a histone deacetylase inhibitor (HDACi) that has shown strong antitumor effects in various types of solid cancers when combined with other traditional chemotherapeutic drugs such as camptothecin and gemcitabine [42–47]. Thalidomide is a glutamic acid derivative and is a classic drug with immunomodulatory properties that inhibits tumor necrosis factor alpha [48–50]. Subsequent studies found that thalidomide has anti-angiogenic, anti-inflammatory, anti-fibrotic, and immunomodulatory effects, and the clinical effectiveness of thalidomide in the treatment of diseases such as refractory Crohn’s disease, lung metastasis, multiple myeloma, hepatocellular carcinoma, and breast carcinoma has been confirmed [51–58]. The CMAP database results provide several potential small-molecule drugs for future NPC therapy. However, *in vivo* and *in vitro* studies are necessary to validate their therapeutic efficacies.

## Conclusion

In summary, by constructing a weighted gene co-expression network with data from the GEO database, our study identified four hub genes correlated with prognosis in NPC tumors and several potential small-molecule drugs for NPC treatment, which may contribute to new insights for NPC therapy. Further studies, including *in vivo* and *in vitro* experiments, are necessary to elucidate the molecular mechanisms of hub genes for future clinical applications.

## Data Availability

The publicly published GEO datasets are available online.

## Abbreviations

NPC: Nasopharyngeal carcinoma; IARC: International Agency for Research on Cancer; ASR: age-standardized; EBV: Epstein-Barr virus; WGCNA: weighted gene co-expression network analysis; GEO: Gene Expression Omnibus; RMA: Robust MultiArray Averaging; GS: gene significance; ME: module eigengene; MM: module membership; GO: Gene ontology; KEGG: Kyoto Encyclopedia of Genes and Genomes; ROC: receiver operating characteristic; CMAP: Connectivity map; CRIP1: cysteine-rich intestinal protein 1; KITLG: KIT ligand; MARPK1: microtubule affinity regulating kinase 1; PGAP1: post-GPI attachment to proteins 1; HDACi: histone deacetylase inhibitors.

## Highlights

1. CRIP1, KITLG, MARK1 and PGAP1 have excellent diagnostic efficiency in NPC.
2. Four prognostic biomarkers and several candidate small-molecule drugs for NPC.
3. Ten small-molecule drugs might provide potentially therapeutic goals for NPC.
